# Bacterial Leakage Testing in Dentistry: A Comprehensive Review on Methods, Models, and Clinical Relevance

**DOI:** 10.1155/sci5/8197293

**Published:** 2025-10-28

**Authors:** Niher Tabassum Snigdha, Mohmed Isaqali Karobari

**Affiliations:** ^1^Department of Dental Research, Saveetha Medical College and Hospital, Saveetha Institute of Medical and Technical Sciences, Saveetha University, Chennai 602105, Tamil Nadu, India; ^2^Department of Conservative Dentistry and Endodontics, Saveetha Dental College and Hospitals, Saveetha Institute of Medical and Technical Sciences, Saveetha University, Chennai 600077, Tamil Nadu, India; ^3^Department of Restorative Dentistry & Endodontics, Faculty of Dentistry, University of Puthisastra, Phnom Penh 12211, Cambodia

**Keywords:** bacterial infection, bacterial leakage test, dental, dental leakage, dental material, root canal obturation, root canal preparation, tooth penetration

## Abstract

**Background:**

The marginal gap increases the rate of bacterial leakage and treatment failure; the measurement of the marginal gap is questionable. The literature revealed that when bacteria get trapped within the smear layer, they can multiply and re-contaminate the root canal system, leading to treatment failure.

**Methodology:**

A literature search was carried out in multiple databases, including PubMed, Web of Science, Scopus, and ScienceDirect, with the help of MeSH terms such as Bacterial infection, Bacterial leakage, Dental leakage, Dental material, Root canal preparation, Root canal obturation, and Tooth penetration.

**Result:**

The initial result of the search showed 2252 articles that were relevant enough. However, only 26 articles were eligible based on inclusion criteria.

**Conclusion:**

The bacterial leakage test can evaluate one of the most essential properties of dental material: sealing ability. The sealing ability of a dental material can prevent bacterial ingestion and reduce the treatment failure rate. This study discussed all the steps of the bacterial leakage test in detail. This study can help the researcher plan and run a bacterial leakage study successfully, and the results can help clinicians choose the best sealing material for the clinical scenario.

## 1. Introduction

The bacterial leakage test is the most frequently used procedure to evaluate the sealing ability of restorative materials in dentistry. The bacterial leakage test is designed to assess bacterial penetration through restoration material, which may be undetectable clinically. Bacterial leakage is a significant factor responsible for failure in dental treatment [1]. The definition of microleakage in dentistry describes the marginal gap around the dental restoration in the tooth surface or structure, which further allows the diffusion of oral fluids, molecules, ions, and bacteria. Microleakage is mainly found at two levels: one at the microlevel, known as bacterial leakage, and another at the nano-level, known as nano leakage [1]. Previous studies have shown that if cariogenic bacteria get access through the restorative material inside the tooth, it causes further infection. Over a hundred years, microleakage has affected clinical treatment outcomes (American Association of Endodontics, 2002). However, the measurement of the marginal gap is questionable [1]. However, a more significant marginal gap increases the bacterial leakage rate and treatment failure [2]. The literature revealed that when bacteria get trapped within the smear layer, they can multiply and re-contaminate the root canal system, leading to treatment failure [1]. Antony et al. [3] used a bacterial leakage model over 6 weeks to evaluate the sealing ability of Biodentine, ProRoot MTA, and EndoSequence RRM putty as a retrograde restoration. *Enterococcus faecalis* was used in this study. Over 6 weeks, ProRoot MTA showed the highest turbidity.

On the other hand, Biodentine and EndoSequence RRM putty did not show any significant difference [3]. Singla and Panghal [4] used a bacterial leakage model using *E. faecalis* to evaluate the sealing ability of three bioactive obturation materials (Endosequence BC sealer, Endoseal MTA, and GuttaFlow bioseal). After 9 weeks of bacterial leakage tests, Endosequence BC sealer and GuttaFlow bioseal became better sealing materials [4].

Dye penetration has been a commonly used microleakage test in dentistry for decades. The dual-chamber bacterial leakage test has recently gained popularity due to its more accurate results. The dual-chamber or split-chamber bacterial leakage model mainly consists of two different chambers, one containing the bacterial suspension and tooth sample (upper chamber) and another one containing the brain heart infusion [5] solution (lower chamber) [6]. Both chambers are adjusted together, and the bacteria pass through the tooth sample. It can be detected in the BHI solution using conventional Gram staining or biochemical testing. The result of bacterial leakage normally correlated with scanning electron microscope (SEM), PCR, or micro-CT, depending on the study protocol [6]. However, P. Datta and S. S. Datta reported in their review article that the results of in vivo studies are less successful than those of in vitro studies performed using dye leakage tests [7]. Yanpiset et al. run a bacterial leakage study on root canal sealers using bioceramic sealer, AH Plus, and bioceramic-impregnated GP. A bacterial leakage study was conducted for 60 days. However, 20%–45% of samples leaked between 42 and 52 days. However, no significant difference between groups was found; even micro-CT reported minimal gap and void in all groups [8].

Dental material with good sealing ability can protect against bacterial leakage in the oral cavity. As these materials are placed inside the oral cavity, they are shown to be nontoxic in the oral environment, biocompatible, and, very importantly, prevent further bacterial ingestion [9]. Bacterial leakage studies can help the clinician choose the most suitable material for the patient scenario. “In vitro” studies conducted using bacterial leakage tests are definitive, as they can be done in human tooth samples; a minimal setup is required for this study, oral bacteria can be selected, and the researcher can mimic the environment [10]. This procedure is less time-consuming, shows accurate and reliable results, and requires fewer human resources and less equipment. The dropout chances of samples in this procedure are pretty low. However, clinical studies of dental materials require many patients and multiple follow-ups for an extended period [10]. During this procedure, many patients drop out. The results of the clinical studies are not entirely based on bacterial leakage, as external or internal factors can cause treatment failure. However, few bacterial leakage studies involving consenting dental material have been published in the last decade [10].

The evaluation of microleakage in dentistry has long been a complicated factor in regulating the success rate of postendodontic treatment. However, the current scenario in this field has been questioned based on the clinical relevance of the conventional microleakage evolution method [11]. Bacterial leakage is linked with endodontic treatment failures and posttreatment difficulties or complications. Multiple studies have represented microleakage, some focusing on addressing bacterial leakage [9]. This review aims to fill this gap by synthesizing existing research on bacterial leakage tests, providing a comprehensive understanding of their relevance and application in endodontics. Bacterial leakage can be avoided by focusing on and improving the endodontic treatment protocol. The current study will highlight the best bacterial leakage method and its protocol, followed by enhancing the efficacy and reliability of endodontic treatment [9]. As the endodontics field evolves, we must reassess and update our understanding of crucial evaluation methods. This review will contribute to this evolution by critically analyzing bacterial leakage tests, ensuring that endodontic practices remain evidence-based and aligned with the latest scientific advancements. A study by Aljabri et al. [12] was conducted in Makkah City, Saudi Arabia, where they investigated the reasons for RCT failure among 131 patients aged 16 and above. The most common reason for treatment failure is underfilling (71%), along with coronal leakage (42.7%), with premolars reporting the highest failure rate in young patients and more in males compared to females [12].

This study contains all the details of bacterial leakage tests that can help researchers design future studies for new and old dental materials step by step.  Table 1 shows previous studies with sample size, bacterial leakage model, materials used in past years, most commonly used bacteria, sterilization method, duration of the study, including results, and others. In this review, the prebacterial leakage test, during-bacterial leakage test, and postbacterial leakage test are elaborated in detail with pros and cons.

**Figure 1:**
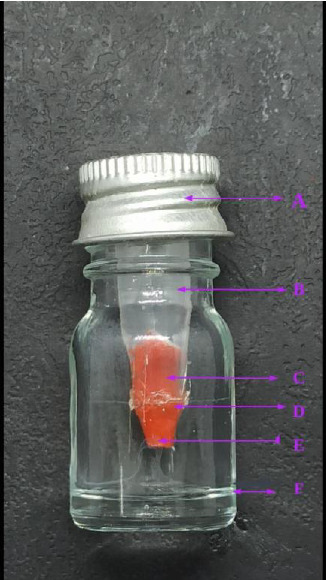
Dual chamber bacterial leakage model (A. screw lid of lower chamber; B. upper chamber; C. sample inside the upper chamber; D. cyanoacrylate; E. protruded sample bottom; F. lower chamber).

In Figure 1, a perfect dual-chamber bacterial leakage model is shown. This model has a plastic upper chamber containing the tooth sample, and the sample is secured using cyanoacrylate adhesive so that the tooth is halfway protruded from the bottom. And a screw-lid glass lower chamber. The current review aims to comprehensively understand dentistry's bacterial leakage tests, which helps with clinical implications.

## 2. Methodology

All the literature in the current study was searched and included from the PubMed, Web of Science, Scopus, and ScienceDirect databases. “Bacterial infection, Bacterial leakage, Dental leakage, Dental material, Root canal preparation, Root canal obturation, Tooth penetration” were used as MeSH keywords for the search. The search was carried out by the two individual reviewers (NT and MIK), and the search strategies are shown in Table 2. The literature search was conducted in the month of November 2024.

Inclusion criteria:

- An in vitro bacterial leakage study was conducted using dental material in extracted teeth.
- Bacterial leakage study using dual chamber leakage model
- Bacterial leakage study conducted in the last decade.
- Bacterial leakage study using *E. faecalis.*

Exclusion criteria:

- Articles are not in the English language.
- Articles from the last 10 years.
- Studies used bacterial leakage tests apart from the dual chamber leakage model.
- Bacterial leakage studies conducted apart from *E. faecalis.*
- Articles from books and conferences, articles irrelevant to the topic.

**Figure 2:**
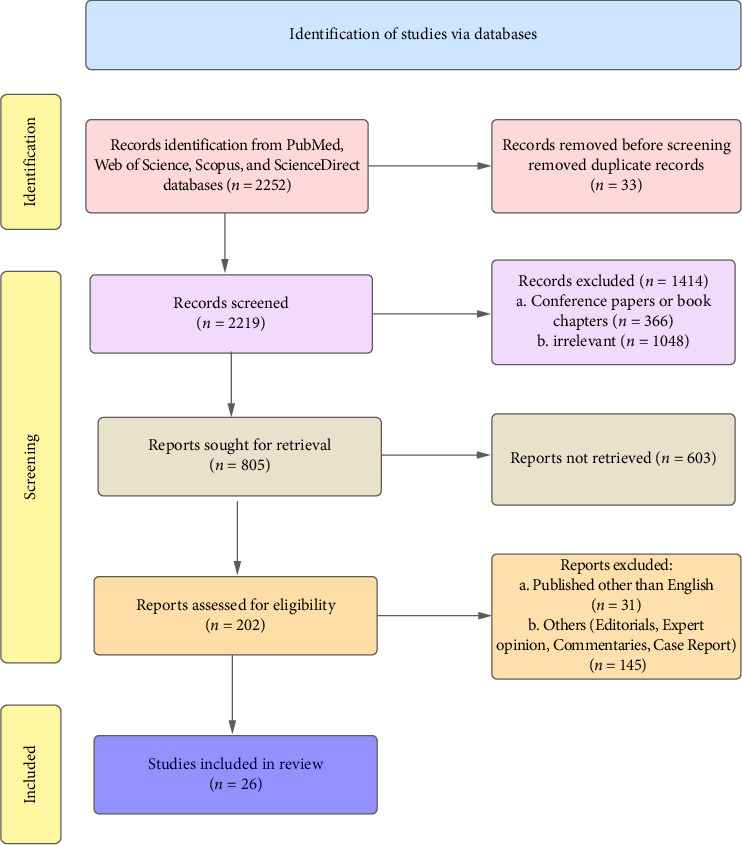
PRISMA flowchart demonstrating the process of selection of articles from multiple web sources.

The full length of all articles was not found. Both revisers went through all the articles, using electric and manual searching. The final articles of the study were selected in 2 stages. In the 1^st^ stage, articles were selected based on titles and abstracts relevant to the current research. From the primary search, a total of 2252 articles were found. Thirty-three articles were removed due to duplication using “endnote.” For the 2^nd^ stage, 2219 articles were left. Articles not in English, articles from books and conferences, articles irrelevant to the topic, and a few articles that would be retrieved, including a few others, were excluded (Figure 2). Both of the reviewers thoroughly read all the articles.

## 3. Results and Discussion

Finally, 26 articles were suitable for the current comprehensive review. Most articles on bacterial leakages are from the last decade of dentistry. A dual-chamber bacterial leakage model is used in all these studies. This study's experiment, or bacterial leakage test period, varies from 25 to 120 days. The sample size varies from 30 to 150, depending on the study requirements. Multiple materials have been used to compare the sealing ability of the material. This study uses different types of material, such as root-end filling material, root canal sealer, coronal barrier, apical plug, pulp dressing, and perforation repairer material. Commonly, the bacteria used in all these studies was *E. faecalis.* The assembling of the dual-chamber model is almost the same in every study, but the sterilization technique of the model is different. The turbidity test of BHI broth of the lower chamber throughout the experiment is standard in most Gram staining experiments. However, the experiment's outcome varies depending on the material and the purpose of the material in that specific study (Table 1).

### 3.1. Bacterial Leakage Model

The dual-chamber bacterial leakage model was first used by Torabinejad et al. [13]. Following this model, many studies have been conducted to date. The bacterial leakage model consists of two upper and lower chambers.

#### 3.1.1. Upper Chamber

After sample preparation according to study requirements, the samples were coated with two layers of nail varnish [3]. To avoid bacterial leakage through dentinal tubules, a plastic tube was taken and cut to place the tooth sample. The tooth sample is placed so the sample is halfway protruded through the tube. Several studies have applied external heat for better adaptation [14]. Cyanoacrylate adhesive is then placed between the tooth and tube to secure the sample and prevent the upper chamber from fluid leakage. A few studies also use sticky wax and hot glue [15]. Few studies used another layer of nail varnish as a final coat [14]. The main objective of the upper chamber is to secure the sample in its position and hold the bacterial broth without any external leakage. The upper chamber is the most crucial part of the bacterial leakage model, as it contains a significant model [16]. The upper chamber contains multiple elements such as tooth samples, nail varnish, adhesive, and plastic; sterilization procedures can hamper any elements. The selection of a sterilization method is essential to secure all the elements [16]. Lertmalapong et al. used a microcentrifugal tube as the upper chamber in his bacterial leakage model [14]. H. A. Buurma and B. J. Buurma used a cutoff plastic pipette tube for the upper chamber and a test tube for the lower chamber to build a bacterial leakage model for this study, where he checked the effect of the smear layer after root canal obturation using a zinc oxide eugenol-based sealer [17].

#### 3.1.2. Lower Chamber

A simple, clear glass bottle is selected for the lower chamber. So that the upper chamber can be easily placed inside it [8], and the lower chamber can contain BHI broth [10], Phenol Red Broth [13], or some other suspension that can cultivate bacteria. However, BHI broth is a commonly used suspension for lower chambers. A clear glass bottle is essential in a few studies because an indicator has been added to the BHI broth. After all, color change in BHI broth indicates or confines bacterial leakage. However, in most studies, a clear glass bottle is unnecessary [16]. However, gamma radiation sterilization can change the color of the glass bottle. Fransen et al. [18] used a 20-clear glass vial in this bacterial leakage study and added an indicator to the BHI broth. The purple color of BHI broth indicated no bacterial leakage, while the yellow color of BHI broth [18]. Ramazani and Sadeghi [19] used a bacterial leakage test to seal the ability of MTA, CEM, and Biodentine as a furcation repair material. Their bacterial leakage model used a 10 mL glass bottle with a lower chamber. After 40 days, MTA and Biodentine showed the most promising results [19].

### 3.2. Sterilization Process of Bacterial Leakage Model

The bacterial leakage model consists of multiple elements such as a tooth sample containing dental material, nail varnish, a plastic tube, cyanoacrylate adhesive, a glass bottle, and others. In different studies, different sterilization methods have been used [20]. Antony et al. placed the whole bacterial leakage model inside the ultraviolet chamber for sterilization [3]. Ultraviolet sterilization is an excellent option to sterilize the dual-chamber model. To eliminate all the bacteria, UV light must set a specific weavelength [21]. Yanpiset et al. sterilized the upper chamber through ethylene oxide gas, and the lower chamber was placed inside the autoclave for 20 minutes [8]. In some studies, BHI broth is poured into a lower chamber and autoclaved for 20 min [14, 15]. Autoclave is a frequently used sterilization procedure in dentistry, but it uses a very high temperature to sterilize, which can lead to distortion of the upper chamber as it contains multiple elements. Gamma radiation sterilization is also a good option, as it can simultaneously enter the bacterial leakage model, including the packaging [22].

In a previous bacterial and dye leakage study by Isabelle Elias et al. (2010) using AH Plus and GuttaFlow. He used 25 KGy of gamma radiation to sterilize the bacterial leakage model. High radiation and extended sterilization periods can also distort the cyanoacrylate adhesive. It is advisable to use low radiation and a short sterilization period [20]. Emami et al. compared the apical sealing ability of gutta-percha in two different stages. His study used ethylene oxide gas for 24 h to sterilize the bacterial leakage model [23]. Ethylene oxide gas sterilization is suitable for heat-sensitive material and does not cause any biological change in the product. However, it does have evidence of crossing multiple layers and sterilizing [24].

### 3.3. Bacteria Commonly Used in Leakage Model

*E. faecalis* is a Gram-positive anaerobic bacterium with extreme survival ability even inside the dentinal tubule and root canal system. *E. faecalis* is the most frequently used bacteria in bacterial leakage tests in dentistry [25]. *E. faecalis* enters the tooth through microleakage and causes a high secondary infection rate [26]. The prevalence of *E. faecalis* is very high in saliva and endodontic infections that need retreatment [25]. *E. faecalis* is highly relevant in persistent infection, well-characterized as well as extremely resilient, behavior reproducible, concentrated, high strain, detection efficacy is precise and experimental control is strong as well as reproducible models compared to any other clinical or polymicrobial strains [26, 27]. However, *E. faecalis* is easily cultured and maintained in the laboratory.

*Streptococcus mutants* [17], *Proteus mirabilis* [18], *Staphylococcus epidermidis* [28], multiple bacteria [29], and human saliva [30, 31] are also used in previous studies. Borges et al. [29] checked the sealing ability of ProRoot MTA, AH-Plus, and SuperEBA as root-end filling material. In their bacterial leakage model, they introduce multiple bacteria (*E. faecalis* + *Pseudomonas aeruginosa* + *Staphylococcus aureus* + Candida *albicans* + *Bacillus subtilis*). After 60 days of the experiment, ProRoot MTA and SuperEBA became better sealing materials as apical plugs [29]. In their study, Oliveira et al. [30] treated teeth with intracoronal posts without crowns and exposed them to human saliva. After 40 days, he recommended the treatment procedure due to less bacterial leakage [30].

### 3.4. Bacterial Leakage Test Procedure

After sterilization, BHI broth is poured into the lower chamber, and the bacterial leakage model is placed inside the incubator at 37°C and 100% humidity to maintain sterility [15]. The sample should be 2 mm immersed in the lower chamber's BHI broth. The preselected bacteria is then collected from the lab or cultured in agar media [32]. Before the experiment, the bacterial colony is collected, rehydrated in BHI broth, stored, and incubated for 24 h at 37 °C and 100% humidity. With the help of a McFarland densitometer, the bacterial broth is then adjusted to the density of 0.5 McFarland standard [14]. The bacterial broth is then poured into the upper chamber in a preselected ratio (500 μL) so that the sample is fully immersed. All the samples are placed inside the incubator during the experiment to avoid contamination and maintain sterility [14]. It is essential to follow every step, as this procedure has a high risk of contamination.

**Figure 3:**
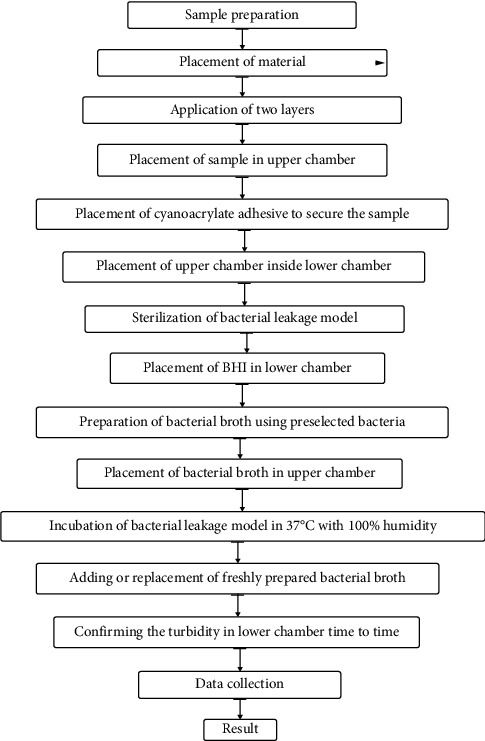
Flowchart of a bacterial leakage test.

The bacterial broth of the upper chamber is replaced by freshly prepared bacterial broth, or freshly prepared bacterial broth is added in the upper chamber from time to time to confirm bacterial viability. The BHI broth of the lower chamber is checked on preselected dates to ensure the bacterial leakage time for a specific period [32]. Prithviraj et al., in their bacterial leakage study, replaced the bacterial broth of the upper chamber every 7 days and checked the turbidity of the lower chamber every day for 30 days [33]. Jahromi et al., in their bacterial leakage study, added bacterial broth in the upper chamber every 2 days and checked the turbidity of the lower chamber every day for 75 days [15]. The bacterial leakage procedure is shown in Figure 3 as a flowchart.

### 3.5. Methods of Confirming the Bacterial Leakage

Confirming the bacterial leakage in the dual-chamber model is the most crucial step of the experiment; the outcome depends on it. Gram staining is the most used method of confirming bacterial leakage or the presence in the broth of the lower chamber. Gram staining is a widely used and essential technique in microbiology to detect Gram-negative and Gram-positive organisms [34]. Gram staining is considered a primary diagnostic test. The benefits of the Gram staining procedure are that it is very cost-effective, no special equipment is required, and the result can be easily diagnosed [35]. For Gram staining, a strict protocol needs to be followed. The procedure is time-specific and time-consuming because all the steps must be done in a specific any error in the timing can lead to false results. After Gram staining, the slides must be checked under a light microscope from 40x to 200x magnification to identify the bacteria [34]. The slide needs to be entirely checked before concluding.

However, nonsterile sample collection can cause contamination. Eskandarinezhad et al. [36] had 40 samples in their bacterial leakage study; they performed Gram staining daily to ensure the bacterial leakage of *E. faecalis.* Lertmalapong et al. [14] used 150 samples in their bacterial leakage study and used Gram staining to confirm the leakage. A few other not-so-popular methods are used for detecting bacterial leakage, such as the culture of the broth of the lower chamber in blood agar media or placing an indicator in the bacterial broth of the lower chamber [16]. Yanpiset et al. [8] used only Gram staining to confirm the leakage in their bacterial leakage study. In this study, authors evaluated the sealing ability of root canals obturated with GP, BCC, BCS, or AH Plus sealers. Ninety-two distobuccal roots of maxillary molars went through a bacterial leakage test for 60 days. And after the bacterial leakage test, no significant difference was found [8]. Singla and Panghal [4] used three bioactive obturation materials and evaluated the sealing ability. GuttaFlow, Endosequence BC sealer, and Endoseal MTA were used in this study as obturation material. A total of 76 teeth underwent bacterial leakage tests for 9 weeks, and during this period, bacterial leakage was detected using Gram staining. After the experiment, GuttaFlow and Endosequence BC sealer obturation material showed better sealing ability [4].

Nowadays, biochemical tests are also used in parallel to confirm bacterial leakage. Catalase test, bile esculin test, PYR. Bile esculin is considered a selective test for *E. faecalis* and is used as an initial detection method, where, after 24 h, black deposits appear to confirm bacterial presence [37]. Eskandarinezhad et al. used a bile esculin test along with PYR, catalase, a 6.5% NaOCl growth test, and an optochin disk to confirm the bacterial leakage after Gram staining in his bacterial leakage study. In this study, he evaluated the sealing ability of MTA with nanosilver and MTA without nanosilver as a root end-filling material. After the experiment, MTA without nanosilver reported better sealing ability [36]. Abdulrazzaq and Al-Nasrawi did a comparative study to check the antimicrobial effect of 17% EDTA, MTAD, and 3% NaCl in root canal therapy in primary teeth against *E. faecalis.* In this study, he infected the samples for 2 weeks, followed by irrigation and incubation in BHI broth. An esculin test was initially performed to detect the bacterial bile. After the experiment, all the irrigation material resulted in a complete absence of bacteria [37].

The catalase test is a procedure where bacteria or organisms, both Gram-negative and Gram-positive, are identified due to enzyme production. Usually, the bacteria are isolated on a glass slide, and a few drops of H_2_O_2_ are placed on the slide [5]; if the bacteria produce bubbles, that proves catalase positive. Depending on the bubble formation, it is determined that the bacteria is catalase-positive or catalase-negative. Catalase-positive bacteria are *S. aureus,* and catalase-negative bacteria include *Clostridium perfringens* and *E. faecalis* [5]. As *E. faecalis* is the most commonly used bacteria in bacterial leakage tests, *E. faecalis* is catalase negative. A PYR test is performed to confirm *E. faecalis*.

The pyrrolidonyl arylamidase (PYR) test is mainly used for *E. faecalis* [38]; it has been quite popular in recent studies. The PYR test takes less than 5 minutes to show results and is straightforward. The *E. faecalis* PYR test is considered more reliable, has better quality, and has less chance of contamination than Gram staining. To perform the PYR test, the bacterial broth must be incubated for 18 to 24 h; only then does the PYR kit provide a more accurate result [38]. Before performing the test, the PYR kit must be kept at room temperature for a few minutes. The PYR disc needs to get enough bacterial broth; otherwise, it can influence the result [38]. In most of the studies, all the samples go for Gram staining first, followed by the PYR test to compare both results and confirm the outcome [14]. However, the PYR test is not very popular in the research community, as it is not budget-friendly. Many samples can cut a considerable budget for the PYR test alone. However, the PYR test can be considered one of the best alternatives for Gram staining.

Recent studies confirm or cross-check bacterial leakage results using SEM. This method creates higher magnification, higher resolution images, and more accurate inorganic and organic material visualization. SEM can capture images up to 10,000,00x in the upgraded models [39]. For bacterial leakage tests in dentistry, mostly 2000x magnification images are used, as dentinal tubules are most prominent and better visualized in this magnification, which leads to more accurate results [40]. However, all studies do not require SEM and a good amount of budget to conduct a study with large samples. Snigdha et al. performed PYR as a biochemical test for their bacterial leakage study to evaluate the sealing ability of bioceramic (MTA, Biodentine, and ProRoot MTA) pulp dressing material and SEM to evaluate the marginal adaptation of the materials [20]. Yanpiset et al. use micro-CT in their bacterial leakage study to evaluate the sealing ability of obturation material [8].

### 3.6. Materials Used for Bacterial Leakage

The bacterial leakage model is commonly used *in vitro* studies to check the sealing ability of dental material. In the last decade, many imitated bacterial leakage studies have been conducted in dentistry; most bacterial leakage studies are conducted on root canal sealer material, apical plug material, coronal seal material, perforation repair material, and others. Bioceramics material is frequently used nowadays. Biodentine [14], MTA [3], ProRoot MTA [29], EndoSequenceRRM [3], TotalFill BC RRM, AH Plus [8], CEM [23], zinc oxide eugenol-based sealer [17], SuperEBA [29], IRM [41], AH26 sealer [23], and others are used in previous bacterial leakage studies. However, any material can be used in bacterial leakage. There is no limitation on that depending on study requirements.

Övsay et al. [41] used ProRoot MTA, Biodentine, and IRM as perforation-repair material. He runs a bacterial leakage test for 45 days. ProRoot MTA was the most successful material in perforation management compared to Biodentine and IRM [41]. Nasri and Afkhami reposted their bacterial leakage study using gray ProRoot MTA AgNPs against coronal leakage as an orifice plug. Human saliva was used in the upper chamber of the bacterial leakage model. Ag-MTA was better after 120 days of the experiment than ProRoot MTA [31].

## 4. Limitations

The current review acknowledges few limitations. The variability in methodologies across studies, including differences in test protocols and bacterial strains, may impact the comparability and generalizability of the review findings. Additionally, publication bias and variations in study quality could influence the results. The rapid evolution of bacterial leakage testing technologies and potential language and access barriers to certain studies may further limit the scope of our review. These factors should be considered when interpreting our conclusions.

### 4.1. Clinical Importance and Further Recommendation

Bacterial leakage studies are essential in selecting material with better sealing ability in clinical settings. The results can influence the selection of dental material. Bacterial leakage studies can guide the clinician about the mean leakage time of material, which can reduce treatment failure.

Bacterial leakage studies can be conducted on a broad spectrum of materials. A bacterial leakage test can check the sealing ability of new materials. However, after creating the bacterial leakage model, GAMA radiation can be used as a sterilization method for a specific radiation for a short period. It is claimed to be safer and can be used in multiple varieties of equipment and materials. Although *E. faecalis* is the most commonly used bacteria, as it is commonly found in the oral cavity and root canal system, future researchers should combine multiple bacteria in future studies for more accurate results. For the bacterial leakage confirmation test, it is better to conduct both Gram staining and PYR biochemical tests, as both can support each other's results more accurately. Another test can be conducted to support the experiment. The results of bacterial leakage studies can be confirmed, followed by clinical studies.

## 5. Conclusion

All bacterial leakage tests in dentistry come with some limitations. Each bacterial leakage study differs from another due to the treatment procedure, the material used for the procedure, bacterial selection, sterilization procedure, study duration, detection of bacterial leakage, and correlating the result with further tests. In the long term, bacterial leakage tests can contribute much to clinical dentistry by providing laboratory feedback about dental material, which can play an essential role in preventing treatment failure.

## Figures and Tables

**Table 1:** The characteristics of the included studies.

Reference	Bacterial leakage model	Sample size (tooth)	Duration of experiment (days)	Material used	The purpose of material	Sterilization method		Bacteria used	Bacterial medium used	Bacterial broth (days) replacement		Bacterial leakage confirmatory test	Test supports the result of the bacterial leakage test	Results	Conclusions
						Upper chamber	Lower chamber			Replaced by freshly prepared bacteria	Adding freshly prepared bacteria				
[42]	Dual camber	34	90	MTA and CEM	Root end filling	Ethylene oxide gas	Autoclave	*Enterococcus faecalis*	Muller–Hinton Broth culture medium	3		Cultured in bile esculin agar	Fluid filtration	There was no significant difference between MTA and CEM in	Both bacterial leakage and fluid filtration reported the same result.
[8]	Dual camber	92	60	AH pulse, bioceramic sealer, bioceramic-impregnated gutta-percha cone	Root canal sealer	Ethylene oxide gas	Autoclave	*Enterococcus faecalis*	BHI	3		Gram staining	Micro-computed tomography (CT) analysis.	Around 20 to 40 percent of the samples were reported to be leaked within 42 to 52 days, and the micro-CT result showed the minimal gap is less than 1 percent for all groups.	There was no significant difference found among the materials.
[14]	Dual camber	150	75	ProRoot MTA, Biodentine, TotalFill BC RRM paste, TotalFill BC RRM putty, and RetroMTA	Apical plug	Ethylene oxide gas	Autoclave	*Enterococcus faecalis*	BHI		1	Gram staining and biochemical tests	SEM	After the experimental period, the samples that did not leak are Biodentine (4 mm) 70%, ProRoot MTA (4 mm) 50%, and TotalFill BC RRM putty (3 and 4 mm) 50%.	The 3- and 4-mm Biodentine and TotalFill BC RRM putty groups and the 4-mm ProRoot MTA had better sealing ability and marginal adaptation.
[43]	Dual camber	30	60	RelyX Luting Plus, Maxcem Elite, and conventional acid-base reaction glass ionomer cement.	Luting agent	Ethylene oxide gas		*Enterococcus faecalis*	Trypticase soy broth (TSB)	5		Cultured in blood-agar plates and Gram staining		RelyX Luting Plus cement microleakage scores are lower than Maxcem Elite and Ketac Cem	RelyX Luting Plus cement is reported to be a better luting agent.
[44]	Dual camber	40	60	ACTIVA BioACTIVECEMENT/BASE/LINER, FujiCem2, Embrace WetBond, and zinc phosphate cement.	Luting agent	Ethylene oxide gas		*Enterococcus faecalis*	TSB	5		Cultured in blood-agar plates and Gram staining		ABC showed leakage on Days 58, 59 and no leakage in 8 samples. FC2 showed on Days 58, 60 and 8 samples did not leak. EWB showed leakage on Days 55, 58 and not leaked 7, ZPC showed on Days 12, 14, 18, 21, 22, and only one sample did not leak.	ACTIVA BioACTIVECEMENT/BASE/LINER, FujiCem2 reported the lowest bacterial leakage.
[45]	Dual camber	70	60	Smart-Seal system (hydrophilic), Resilon/Epiphany system (hydrophilic), and AH Plus system (hydrophobic)	Root canal sealer	Ethylene oxide gas	Autoclave	*Enterococcus faecalis*	BHI	7		Gram staining		After 60 days of experiment, bacterial leakage was reported in groups are Smart-Seal System 9, Resilon/Epiphany 10, and AH Plus 18 samples.	Hydrophilic obturation has shown better bacterial resistance.
[46]	Dual camber	64	60	MTA and CEM	Root canal sealer	Ethylene oxide gas		*Enterococcus faecalis*	BHI		3	Catalase, bile esculin, PYR, optochin disk, and 6.5% NaOCl growth tests		The bacterial interventions of this study are MTA 40.68 ± 11.03 and CEM 39.56 ± 9.03.	There was no significant difference, but well-fitted gutta-percha with single-cone obturation is advisable.
[47]	Dual camber	50	120	MTA and GP	Root canal sealer	Placed inside the incubator		*Enterococcus faecalis*	BHI	7		Gram staining		After the duration of the experiment, the bacterial leakage was reported as GP 18.5 days, MTA (8 mm) 93 days, and MTA (4 mm) 95 days.	MTA is considered the best replacement for GP for apical third restoration in a tooth indicated for a post.
[48]	Dual camber	45	70	White and gray mineral trioxide aggregate (WMTA and GMTA), CEM, Portland cement	Root-end filling	Ethylene oxide gas		*Enterococcus faecalis*	TSB		5	Cultured in blood agar, simple agar, bile esculin, and Gram staining	Dye leakage test	Portland cement and CEM reported 100% leakage during the study period, GMTA 58.3%, and WMTA 91.7%.	CEM showed better sealing ability as a root end filling material compared to others.
[15]	Dual camber	70	75	Biodentine, ProRoot MTA, CEM	Apexification or apical plug	Placed inside the incubator		*Enterococcus faecalis*	BHI		2	Cultivated		The highest level of turbidity was recorded in ProRoot MTA 90%, followed by CEM 70% and Biodentine 50%.	Biodentine showed the most promising sealing efficacy.
[36]	Dual camber	70	90	MTA and MTA with nanosilver	Root end filling	Ethylene oxide gas		*Enterococcus faecalis*	BHI		3	Gram staining, catalase, bile esculin, PYR, optochin disk, and 6.5% NaOCl growth test		The mean bacterial leakage time of MTA with nanosilver and MTA is 9.66 ± 14.25 and 30.06 ± 28.67, which reported a significant difference.	MTA without nanosilver showed better sealing ability in the bacterial leakage test
[49]	Dual camber	76	90	AH Plus, MTA Fillapex	Root canal sealer	Ethylene oxide gas		*Enterococcus faecalis*	BHI		3	Gram staining		During the study period, both materials reported a similar amount of leakage, which leads to similar results for both materials.	No significant difference was reported.
[50]	Dual camber	52	42	MTA, calcium silicate cement with zirconium oxide (CSC/ZrO_2_), and zinc oxide eugenol (ZOE)	Root end filling	Ethylene oxide gas		*Enterococcus faecalis*	BHI	7		Gram staining	Solubility test	The solubility test was conducted for 7 days. And ZOE was the most soluble reported. The bacterial leakage score for MTA, CSC/ZrO_2_, and ZOE was 4.5 ± 1.0, 4.5 ± 0.9, and 3.1 ± 1.1	In this study ZOE reported the most solubility; on the other, hand CSC/ZrO_2_ and MTA showed better sealing ability.
[51]	Dual camber	70	90	AH26, MTA Fillapex, and MTA-PG sealer (dry and wet)	Root canal sealer	Autoclave		*Enterococcus faecalis*	BHI		1			During the experimental period the turbidity was reported as 46.7% in MTA-PG dry, 80% in MTA-PG wet, 66.7% in MTA fillapex, and 93.3% in AH26.	MTA-PG [52] sealer reported superior sealing ability in the bacterial leakage test.
[53]	Dual camber	60	45	MTA, CEM, and Biodentine	Coronal barrier	Ethylene oxide gas		*Enterococcus faecalis*	BHI		2	Cultured in a medium of blood agar		Each group had 16 samples. Bacterial leakage was reported in 11 samples of MTA, 12 samples of Biodentine, and 9 samples of CEM.	Biodentine and CEM showed a better result as a coronal barrier in the bacterial leakage test.
[54]	Dual camber	90	90	Laser irrigation with MTA and MTA	Retro gets filling	Ethylene oxide gas		*Enterococcus faecalis*	BHI		3	Cultivated in blood agar culture medium	SEM	After 90 days of experiment, the mean leakage time of MTA with laser is 77.68, and MTA without laser is 51.60	MTA without laser irrigation showed better sealing ability compared to MTA with laser irrigation.
[55]	Dual camber	142	90	MTA Fillapex, Apatite Root Canal Sealer, and AH26 sealers	Root canal sealer	Ethylene oxide gas		*Enterococcus faecalis*	BHI		Everyday	Indicator		After the experiment, the bacterial leakage percentage was MTA Fillapex—44%, AH26—36.3%, and Apatite Root Canal Sealer—56.8%.	AH26 sealer reported significantly higher sealing ability compared to others.
[19]	Dual camber	61	90	MTA, CEM, and Biodentine	Furcation Perforation repair material	Ethylene oxide gas		*Enterococcus faecalis*	BHI		2	Indicator		Within the experiment time, the bacterial leakage occurred with MTA Fillapex 50%, AH26 36.3%, and Apatite Root Canal Sealer 56.8%.	There was no significant difference detected between the three perforation repair materials.
[56]	Dual camber	83	70	MTA, CEM, and IRM	Root end filling	Ethylene oxide gas		*Enterococcus faecalis*	BHI		Everyday	Special culture media		The presence of bacterial leakage was detected in this study: MTA—7%, IRM—13%, CEM—13%. Although the presence of leakage is less in MTA but, it is not significant.	All the material used in this study has equal sealing ability for bacterial leakage.
[57]	Dual camber	50	90	iRoot SP and ProRoot MTA	Root end filling	Ethylene oxide gas	Autoclave	*Enterococcus faecalis*	BHI		1	Indicator and cultured in a medium of blood agar		ProRoot MTA showed bacterial leakage in between 30 and 72 days, and iRoot SP showed it in between 51 and 69 days.	iRoot SP showed better sealing ability compared to ProRoot MTA.
[4]	Dual camber	76	53	Endosequence Bioceramic sealer, Endoseal MTA, and GuttaFlow bioseal	Root canal sealer	Ethylene oxide gas	Autoclave	*Enterococcus faecalis*	Phenol red broth	7		Gram staining		After the duration of the bacterial leakage test, the mean leakage was Endosequence 6.727%, BC sealer 5.045%, and Guttaflow bioseal 6.182%.	Endosequence, BC sealer, and Guttaflow bioseal sealer reported promising apical seals with the single-cone technique compared to Endoseal MTA.
[58]	Dual camber	100	120	White Portland cement and white MTA	Root end filling	Ethylene oxide gas		*Enterococcus faecalis*	BHI		7	Culture media		In this study three different mixing methods were used to prepare the material: ultrasonic, conventional, and amalgamator. The leakage time was 78.5 ± 13.46, 80.2 ± 13.64, and 84.667 ± 11.42 for white Portland cement and 66 ± 13.32, 49.13 ± 12.96, and 69.07 ± 11.5 for white MTA.	After the bacterial leakage test, no significant difference was found.
[59]	Dual camber	70	120	White MTA, MBPc, and CPM	Apical plug	Ethylene oxide gas		*Enterococcus faecalis*	BHI			Gram staining		White MTA showed leakage in 7 samples, CPM showed in 4 samples, and MBPc showed in only 1 sample	MBPc was reported to have less bacterial leakage compared to CPM and WMTA.
[20]	Dual camber	55	25	MTA, Biodentine, and ProRoot MTA	Pulp dressing	Gamma radiation		*Enterococcus faecalis*	BHI	1		Gram staining, PYR	SEM	In this study, MTA, Biodentine, and ProRoot MTA are used as coronal pulpotomy pulp dressing material. After the bacterial leakage test, survival time for Biodentine was 11.6 ± 0.804 days, MTA was 17.667 ± 1.413, and PrRoot MTA was 20.867 ± 1.529 days. SEM showed the same alignment of results as well.	ProRoot MTA showed better sealing ability and marginal adaptation.
[60]	Dual camber	55	30	Biodentine, EndoSequence Root Repair Putty, and MTA repair high plasticity	Apical Barrier	Ultraviolet chamber		*Enterococcus faecalis*	BHI	1		UV spectrophotometer		Biodentine, EndoSequence Root Repair Putty, and MTA repair high plasticity are used as apical plug material in immature permanent teeth. After 30 days of bacterial leakage tests, EndoSequence Root Repair Putty showed the least leakage, followed by MTA repair with high plasticity and Biodentine.	EndoSequence Root Repair Putty reported better results compared to MTA repair, high plasticity, and Biodentine.
[61]	Dual camber	66	60	Biodentine, Z 250, Cimpat, Maxxion R, Flow, and IRM	Coronal seal	Ethylene oxide gas		*Enterococcus faecalis*	BHI	3		Gram staining, catalase test		After the duration of the experiment, the mean values of the materials are Biodentine 56.90 days, Z250 Resin 54.90 days, and White Cimpat 53.30 days. Maxxion R 51.30 days, Flow Resin 50.7 days	Biodentine showed better results, followed by Z250 Resin, White Cimpat, Maxxion R, and Flow Resin

**Table 2:** Sources and searched strategies information.

Database	Search strategies	Results
PubMed	((((((((Bacterial infection [Title/Abstract]) AND (Bacterial leakage [Title/Abstract])) OR (Dental leakage [Title/Abstract])) OR (Dental material [Title/Abstract])) OR (Root canal preparation [Title/Abstract])) OR (Root canal obturation [Title/Abstract])) AND (Tooth penetration [Title/Abstract])) AND (In vitro technique [Title/Abstract])) OR (Dental marginal adaptation [Title/Abstract])	74
Scopus	TITLE-ABS (bacterial AND infection) OR TITLE-ABS (bacterial AND leakage) AND TITLE-ABS (dental AND leakage) OR TITLE-ABS (dental AND material) OR TITLE-ABS (root AND canal AND preparation) OR TITLE-ABS (root AND canal AND obturation) OR TITLE-ABS (tooth AND penetration) AND TITLE-ABS (ion AND vitro AND technique) OR TITLE-ABS (dental AND marginal AND adaptation)	35
Web of Science	((((((((AB=(Bacterial infection)) OR AB=(Bacterial leakage)) AND AB=(Dental leakage)) OR AB=(Dental material)) OR AB=(Root canal preparation)) OR AB=(Root canal preparation)) OR AB=(Tooth penetration)) AND AB=(In vitro technique)) OR AB=(Dental marginal adaptation)	1557
ScienceDirect	((((((((AB=(Bacterial infection)) OR AB=(Bacterial leakage)) AND AB=(Dental leakage)) OR AB=(Dental material)) OR AB=(Root canal preparation)) OR AB=(Root canal preparation)) OR AB=(Tooth penetration)) AND AB=(In vitro technique)) OR AB=(Dental marginal adaptation)	586
Total		2252

## Data Availability

The authors have nothing to report.
